# Circular manufacturing of chitinous bio-composites via bioconversion of urban refuse

**DOI:** 10.1038/s41598-020-61664-1

**Published:** 2020-03-13

**Authors:** Naresh D. Sanandiya, Christoph Ottenheim, Jun Wei Phua, Augusta Caligiani, Stylianos Dritsas, Javier G. Fernandez

**Affiliations:** 1 Singapore University of Technology and Design, 8 Somapah Road, Singapore, 487372 Singapore; 2 Institute of Chemical and Engineering Sciences, 1 Pesek Road, Singapore, 627833 Singapore; 3 Insectta, 60 Jalan Penjara, Singapore, 149375 Singapore; 4 University of Parma, Str. dell’Università, 12, 43121 Parma, PR Italy

**Keywords:** Composites, Mechanical engineering, Environmental impact

## Abstract

Bioinspired manufacturing, in the sense of replicating the way nature fabricates, may hold great potential for supporting a socioeconomic transformation towards a sustainable society. Use of unmodified ubiquitous biological components suggests for a fundamentally sustainable manufacturing paradigm where materials are produced, transformed into products and degraded in closed regional systems with limited requirements for transport. However, adoption is currently limited by the fact that despite their ubiquitous nature, these biopolymers are predominantly harvested as industrial and agricultural products. In this study, we overcome this limitation by developing a link between bioinspired manufacturing and urban waste bioconversion. This result is paramount for the development of circular economic models, effectively connecting the organic by-products of civilization to locally decentralized, general-purpose manufacturing.

## Introduction

As a by-product of human activity, solid waste is an accelerating problem strongly coupled with the global trend of rapid urbanization. Waste generated by urban residents is four times greater than their rural counterpart and with the steady increase of urban population and living standards, global waste production is further amplified. Hence, waste management is one of the most expensive services in municipalities around the world and its continuous need for growth is becoming out of reach for the developing countries while strenuous for the developed ones^1^. Notably, in 2018, the global municipal solid waste was estimated to reach ca.2 billion tons per year. At this rate, it is expected to surpass 3.4 billion tons by 2050^2^, and despite the heightened social awareness of the issue, it will continue to accelerate throughout this century^1^. What progressively becomes clear is that at this pace, piecemeal mitigations may not suffice to exit this vicious spiral. Instead, we need to transition to fundamentally different modes of production and consumption based on materials and processes innately capable of supporting sustainable and circular growth paradigms^3,4^.

Although urban waste varies between countries and seasons, in general it is roughly composed of 60% organic matter from food, vegetables, and garden waste; 30% paper, cardboard, textiles, and other cellulosic materials; 12% plastics; 3% metals; and 3% glass^5^. For non-biodegradable waste streams such as plastics, emphasis is placed on recovery and recycling, while for organic streams, attention is focused on reduction and valorising. As food loss and waste is estimated to be approximately one-third of all food produced globally^6^, considerable efforts are aimed toward valorisation. Valorisation of food waste is primarily performed by bioconversion using microorganisms, enzymes, and animals, aimed at the extraction of proteins for animal or human consumption as well as the production of energy such as methane and biofuels^7^. The black soldier fly (BSF, *Hermetia illucens*) became a popular insect globally for its efficient conversion of a wide variety of organic materials, such as urban and agricultural waste, into biomass^8^. In the average 14 days between egg hatching and the prepupal stage, BSFs process 20 times their own weight in waste^9^, reaching an average weight of 220 mg per individual. Chitin, constituting 6–9% of BSFs’ dry weight, is the second-most abundant organic polymer on earth with annual worldwide bioproduction estimated at 10^11^ tons across every ecosystems^10^. By comparison, the amount of chitin naturally produced in 1 year is equivalent to greater than 300 years of the current worldwide plastic production (i.e., 3 × 10^8^ tons/year^11^). Chitin-producing organisms span across most biological kingdoms, including most heterotrophs used as bio-converters of organic waste (e.g., fungi, arthropods, and annelids), as well as in mollusks and even recently, has been extracted in small amounts from vertebrates^12^. Despite its unparalleled ubiquity, currently, crustacean shells constitute the main source of 25,000 annual tons of industrial chitin, which are a by-product of the fishing industry^13^.

Motivated by the ubiquity and mechanical relevance of chitonous polymers, from an early hypothesis some ten years ago^14^ bioinspired chitinous composites developed rapidly towards general-purpose manufacturing by demonstrating their capability for producing large-scale objects, integrating with additive manufacturing, fabricating within limited energy requirements, retaining costs similar to commodity plastics^15^, and perhaps most critically, being able to maintain seamless integration with the ecological cycles of earth spanning from their renewable sourcing and biological composition to their natural biodegradation^16^. In this study, earlier results on bioinspired chitinous manufacturing of fungus-like adhesive materials (FLAM) are correlated to recent advances on large-scale urban bioconversion, effectively transforming the most abundant types of urban waste into materials for sustainable manufacturing. The process is centered around the first and second most ubiquitous organic polymers on the earth, namely cellulose and chitin. These polymers are produced and degraded in large amounts by living organisms in every habitat, including urban ecosystems, enabling the possibility for adoption worldwide. Coupled with their ability for free-form additive manufacturing, the process presented here allows for the first time a general route to embed manufacturing within its surrounding ecosystem.

## Results and Discussion

**Figure 1:**
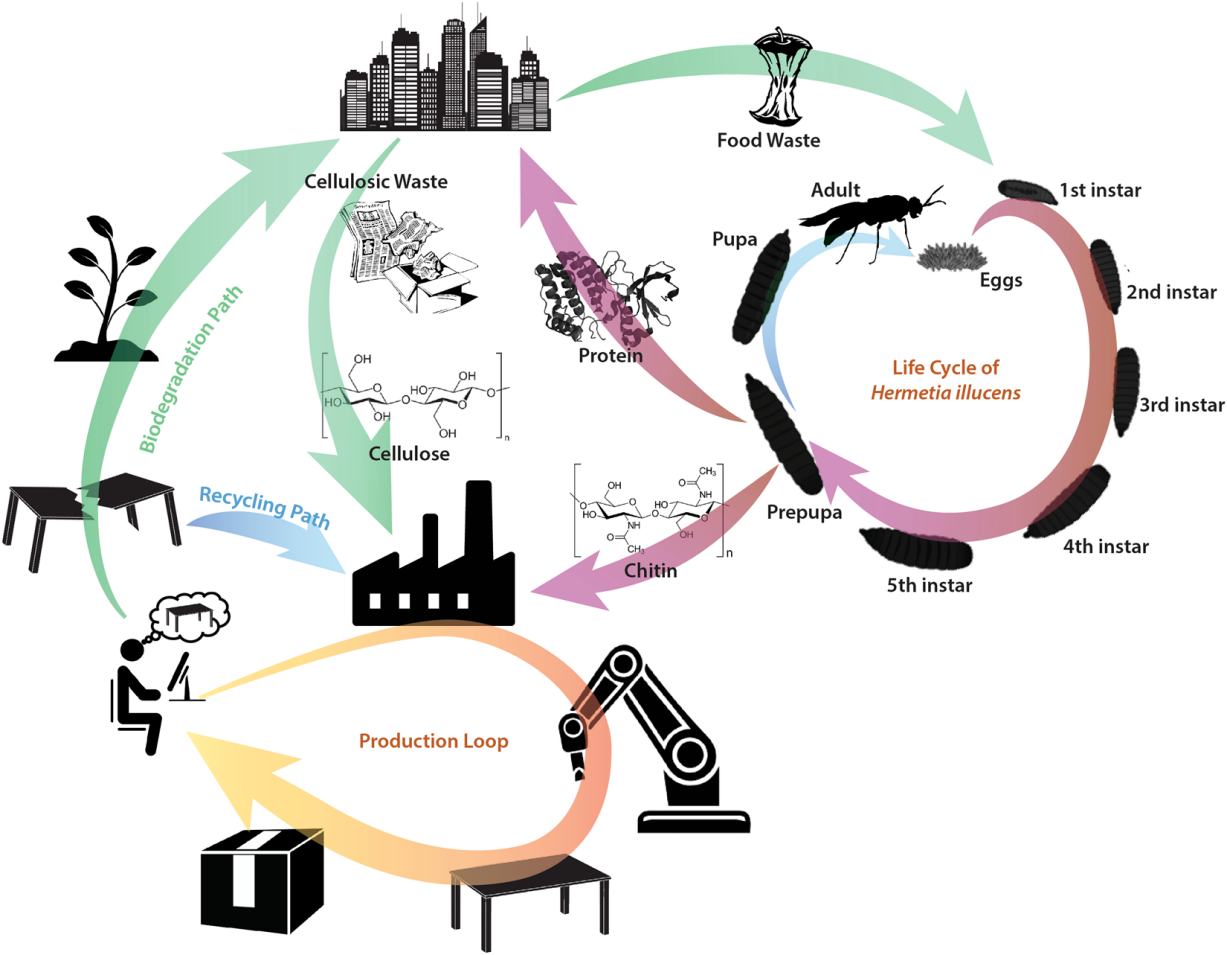
Schematic of the developed closed production loop based on bioinspired chitinous materials and bioconversion in an urban environment. Briefly, food and cellulosic waste are used as the main sources of biomaterials. A large amount of cellulose requiring minimal processing is present in several waste streams, while chitosan is produced by the black soldier fly bioconversion of food waste. Insects are grown through 5 instars and processed at the prepupal stage. Chitosan and cellulose are used without modification to produce fungus-like adhesive materials (FLAM), which are manufactured into any shape by additive manufacturing. The lack of the molecular transformation of natural molecules results also on completely biodegradable objects; hence, waste recovery and processing are minimized, while the same materials can be introduced back into the process in case they are recovered.

**Figure 2:**
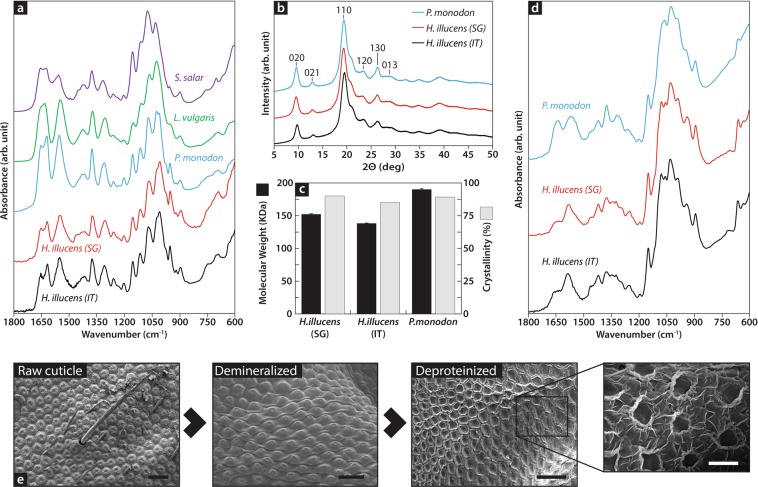
Chitin isolation and characterization from the European and Asian black soldier fly. (**a**) The chitin fingerprint region in the infrared spectrum of the black soldier fly extracted from Atlantic salmon (*Salmo salar*, purple line), common squid (*Loligo vulgaris*, green line), tiger prawn (*Penaeus monodon*, blue line), and black soldier fly from Singapore and Italy (*Hermetia illucens*, red and black lines, respectively) confirms the similarity across sources, regarding different species and geographical origins. (**b**) The X-ray diffraction also confirms the similarity of the crystal conformations between urban-derived chitin and that derived from the current main source of shrimp shells, the latter used in the past to manufacture both 2D and 3D objects and composites. (**c**) Molecular-weight analysis based on viscosimetry and the degree of crystallinity obtained from b also confirm this similarity. (**d**) Additional transformation of chitin into the more deacetylated chitosan for fabrication. The more prominent band observed at 1585 cm^−1^ corresponds to the newly added amine groups. (**e**) Scanning electron micrographs of the different isolation steps involved in the extraction of chitin and the changes induced on the integument of the insect, from the raw insect cuticle (left) to the isolated structural chitin (right).

An overview of the developed process is presented in Fig. 1, where organic refuse from food waste was bioconverted using BSF, grown for 2 weeks through 5 instars, in Italy and Singapore. BSFs were used as they constitute the dominant technology for the bioconversion of organic waste. Italian BSFs were fed with a mixture of agri-food byproducts as fruit and vegetable remains and flour waste from local mills (1:1 w/w) in the town of Ponzano, Veneto region, while Singaporean BSFs were fed with brewery spent grains and okara from the city-state. During the prepupal stage, insects were collected and processed for the separation of fat, proteins, and chitin. Chitin is the main structural component of the cuticle of BSFs, which is present across all instars. Here, it was extracted during the prepupal stage due to its highest protein content which is the main commercial product obtained from the bioprocessing of organic waste. Extraction of chitin focused on the characterization of the polysaccharide toward its application in manufacturing with emphasis on purity rather than optimization of yield, economy of the process, or preservation of other constituents. First, chemical, enzymatic, or stepwise extraction was performed to isolate and/or eliminate non-chitinous components^17^, followed by purification based on previous protocols reported for the isolation of chitin from other species^12^ (Supplementary Fig. 1). Independent of the protocol, region, and diet of the insects, isolated chitin exhibited an infrared fingerprint similar to that of chitin extracted from shrimp, squid, and fish. The amine I band was split into two bands at 1,652 and 1,619 cm^−1^ (Supplementary Tables 1 and 2), respectively, resulting from the two hydrogen bonds in the antiparallel alignment of α-chitin; this result confirmed that the nature of chitin from BSF is similar to that extracted from other arthropods, albeit different from the isomeric form found in squid (i.e., β-chitin, Fig. 2a). This similarity is also confirmed by X-ray diffraction (Fig. 2b), and it is in agreement with the previous characterization data of chitin extracted from other insects, such as bees^18,19^, cicada^20^, and silkworms^21^, as well as previous studies on BSF^22^. Arthropod chitin, including that of insects, is characterized by a high degree of acetylation; hence, strong solvents are required for its dispersion. In contrast, chitin with a deacetylation degree greater than 50% (i.e., chitosan), such as that produced in fungi, can be dispersed in low-concentrated acids due to the introduction of repulsive forces between the easily protonated amine groups. To avoid processing that requires environmentally harmful solvents, insect chitin was further deacetylated to an average degree of deacetylation of 86.82 ± 2.86% (Fig. 2c) using sodium hydroxide and later dispersed in a 1% v/v solution of acetic acid (table vinegar 4–10%).

**Figure 3:**
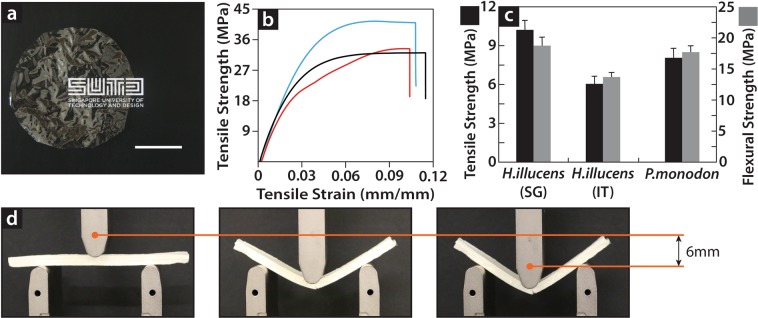
Mechanical test of bio-converted insect chitosan and chitinous composites. (**a**) A chitosan film with a diameter of 12 cm and thickness of 200 µm bio-converted from food waste and placed in front a university logo to reflect the film transparency. (**b**) Mechanical characteristics of chitosan films from bio-converted food waste waste (*P. monodon*, blue line; Singaporean *H. illucens*, red line; Italian *H. illucens*, black line). The obtained results are similar to those previously reported for crustacean shells, highlighting the suitability of this new source to reproduce the manufacturing levels achieved using the latter. (**c**) Mechanical characteristics of cellulose–chitin composites (FLAMs) prepared using urban refuse are similar to those of the previously reported crustacean-based counterpart. The resulting material exhibits mechanical properties within the range of those of soft-wood, high-density polymeric foams, and high-porosity metal foams. (**d**) Stages of the three-point bending test of insect FLAM, form the initial stage (left), right after the elastic region (center), and at breaking (right).

Isolated chitosan (Fig. 2d,e) was characterized and cast into ~250-µm-thick films (Fig. 3a, Supplementary Fig. 3), which were subjected to mechanical tests. These tests represent the first mechanical characterization of chitosan from an insect source to the best of our knowledge. The ultimate tensile strengths of insect chitosan produced by bioconversion in Europe and Asia were 31.86 ± 1.95 and 33.31 ± 1.11 MPa, respectively, with Young moduli of 1.09 ± 0.22 and 1.15 ± 0.05 GPa; these values are in the range of the tensile strength values for shrimp chitin (41.03 ± 3.51 MPa, Young modulus 1.37 ± 0.08 GPa) and commodity plastics (25–35 MPa)^23^ (Fig. 3b). The molecular characterization of bioconverted urban chitosan and its film-forming ability and mechanical characteristics, which are similar to those reported in the past for chitosan extracted from marine sources, highlight the suitability of the material to reproduce all previous results on bioinspired manufacturing with chitinous polymers^24^. In addition, the characteristics of bioconverted insect chitosan are independent of the geographical location and organic substrates (i.e., waste), confirming the global suitability of this biological resource for bioinspired circular manufacturing, independent of the ecology and resources utilized.

Mechanical results obtained for the chitosan films offered encouraging evidence pertaining to their suitability for bioinspired chitinous manufacturing and their potential for widespread applications for producing strong composites and three-dimensional objects. Motivated by this success, its ability to form chitosan–cellulose bioinspired composites, also known as FLAMs, was investigated^15^. FLAMs, which mimic the composition of an oomycetal wall, are currently produced at costs similar to those of commodity plastics and ten times less than those of the cheapest plastic filaments used currently for additive manufacturing (i.e., 1.6$/kg, using 0.7$/kg wood pulp and 12$/kg chitosan). As FLAMs are synthesized using unmodified biomolecules, their production and degradation is seamlessly integrated in the ecological cycles of earth. Here, FLAM was produced using the chitosan from the bioconversion of food waste reported above and cellulose from local waste (i.e., tissue paper and plant matter), and the latter was ground to achieve powder size of 200–500 µm. A stiff composite (E = 0.26 GPa) was obtained, with mechanical characteristics in the range of those of rigid polymer foams, metals foams, and softwoods^25^ and similar to those reported for FLAMs of shrimp and plant origin^15^. Notably, despite the low mechanical differences between the bioconverted-chitosan-derived films and crustacean-derived chitosan films, such differences disappear when they are used to form FLAM.

**Figure 4:**
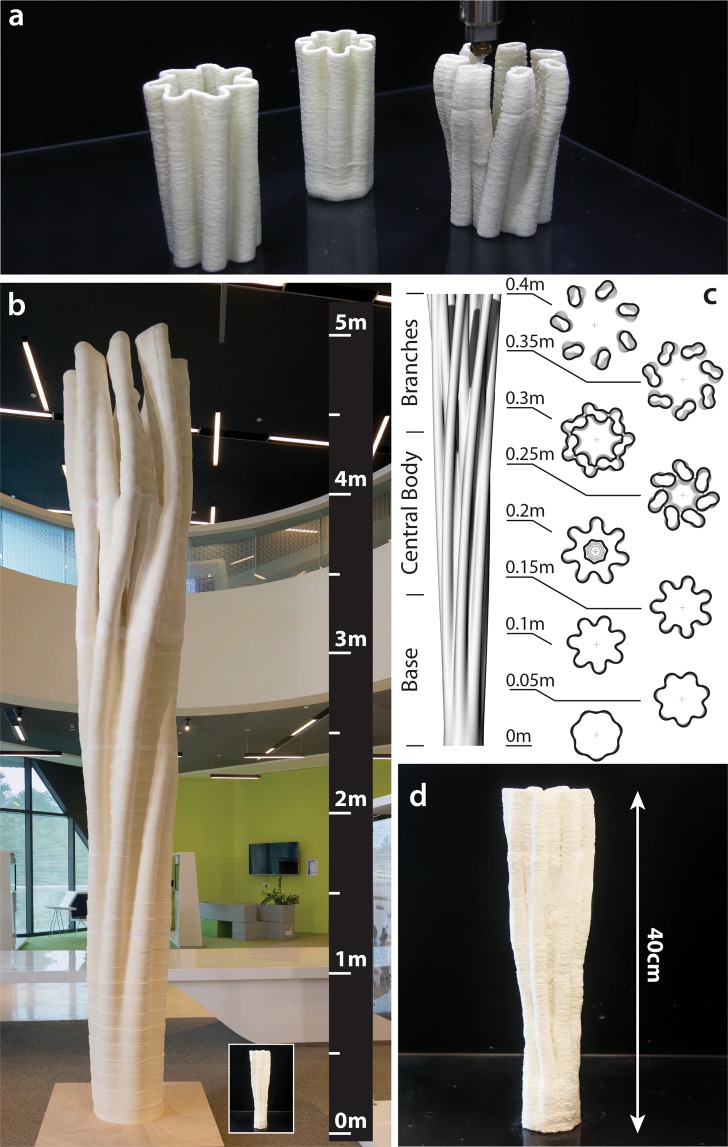
Additive manufacture with bio-converted chitin and cellulose from urban refuses. (**a**) Printing of chitin–cellulose composites derived from urban refuse. The object printed is a replica of “the Hydra”, a geometry that cannot be reproduced by casting/molding techniques. The printing process (Supplementary Movie 1) is performed by segmentation, affording three parts that are joined together using FLAM material. (**b**) Image comparing the original Hydra, which is produced using crustacean chitin and plant cellulose, to the scaled version produced from bio-converted chitin and cellulose from urban waste (inset). The image included four orders of geometrical control from the half-millimeter printing thread used for the scaled version to the 5-m size of the original one. (**c**) Sketch of the internal geometry defining the hydra. The three segments are marked at the left margin. The right side shows the geometrical cross-section at different heights. The diameter of the base is half of that at the top, rendering an unfavorable inverted funnel structure to the item to highlight the mechanical properties of the material. (**d**) Photograph of the insect-based replica of the Hydra.

Material extrusion (ISO/ASTM 52900) was employed for free-form additive manufacturing of waste-based FLAM. FLAM was pumped into a volumetric dispenser, mounted on an industrial articulated robot, via a pneumatic cylinder. Material rheology and process parameters such as the feed, flow rate, and layer height were configured by design of experiment methodology^26^. High-resolution/small-scale models are 3D printed using 1.5 mm nozzle diameter, at 15 mm/s feed rate, 10 ml/min flow rate and 0.75 mm layers. Low-resolution/large-scale objects are 3D printed using 7 mm nozzle diameter, 50 mm/s feed rate, 115 ml/min flow rate and 3.5 mm layer height. Geometries exclusive to additive manufacturing (i.e., unachievable by conventional casting methods) were 3D printed using the high-resolution setup due to limited amount of available material (Fig. 4a, Supplementary Fig. 4). Among the printed models, a key result was a scaled version of the “Natural Composite Pillar”, which is the largest biological object 3D printed today (Fig. 4c,d, and Supplementary Movie 1). This is a noteworthy demonstration of the technology not merely because of the origin of the source material employed and its significance for circular manufacturing models, but also because the technology used in the past to produce the 5 m FLAM prototype, based on crustacean chitin and plant cellulose, is the same used herein to produce a 40 cm tall waste-based scaled version as shown in Fig. 4d. The versatility of the technology is presented when the two objects are placed together (Fig. 4b); from the 0.5 mm post-drying layer definition of the insect replica to the 5 m height of the original crustacean-plant version, there are four orders of magnitude achieved using the same free-form manufacturing system and material.

## Conclusion

In conclusion, we presented a comprehensive study on steps towards developing a new approach to circular manufacturing, spanning from material synthesis using ingredients sourced from urban refuse to creating three-dimensional objects using chitinous-cellulosic biocomposites using contemporary digital manufacturing methods. We showed that a major roadblock, namely chitin equivalent to this predominantly available as fishing industry seasonal by-product, found in limited rural locations, can be sourced from BSF insect bioconversion of food waste at different parts, and potentially every part of the world. Combined with another major source of urban waste, namely cellulose, it can produce biocomposites which can not only address the problem of urban waste but also offer an alternative to plastics. The notion of bioinspired manufacturing, ie., replicating the principles of natural fabrication^27^ such as use of locally available resources, synthesizing complex composites from unassuming ingredients within low-energy environments and creating artifacts fully embedded within their systemic ecology, hold lessons we believe are critical for a new more-creative less-reductionist paradigm, positive steps presented here, towards a more sustainable and circular mode of production and consumption.

## Material and Methods

### Materials

Frozen press-cake of Black Soldier Fly (BSF) prepupae (50% w/w dry mass) was supplied by Insectta, Pte. Ltd., Singapore and referred herein as *H. illucens* (SG). Partially enzymatically or chemically deproteinized and demineralized chitin from BSF was supplied by University of Parma, Italy^17^ and referred as *H. illucens (IT)*. Reagent grades of sodium hydroxide, hydrochloric acid, glacial acetic acid, and sodium acetate were used as supplied by Sigma-Aldrich, USA. Cellulose was obtained from local waste by grinding down tissue paper, printing paper, and plant matter, and homogenized with a sieve to a 200-500 µm particle size. The geometries reported here were produced using the ground tissue paper.

### Extraction of chitin

Extraction of chitin from BSF biomass collected from Singapore was performed by demineralization and deproteinization steps^22^. Demineralization step was carried out by treating pupae cake in 1 N HCl solution(1:10) at room temperature for 2 h to remove minerals and catechols. It was then filtered through It was then filtered through a 100 µ sieve and washed several time with distilled water until the neutral pH of the distilled water. Deproteinization and removal of melanin was performed using 1 M NaOH solution at 80 ^o^C for 12 h. Alkali treatment was repeated two more times to remove proteins completely. The chitin product was filtered and washed with distilled water until the neutral pH followed by drying in an oven set at 40 ^o^C for 12 h and stored in zip lock bag for further use.

### Deacetylation of chitin

Dried chitin isolates were deacetylated in Sodium hydroxide (50% w:w solution, 1:20) at 120 °C for 8 h in a three-necked flask attached with condenser^28^. the samples were filtered and washed several times with distilled water until neutral pH followed by drying in an oven set at 40 ^o^C for 12 h and stored in zip lock bag for further use.

### Molecular characterization and imaging

The surface morphology of samples was examined using a Field Emission Scanning Electron Microscope (FESEM, JEOL, JSM-7600F) at 5 kV accelerating voltage, as previously reported^15^. Samples were gold-coated for 30 s while being mounted onto a carbon tape on an aluminum stub. X-ray diffraction diagrams of chitin and chitosan samples were obtained with a D8 Discover Bruker Diffraction System (XRD), using nickel-filtered CuKα radiation (λ = 0.15418 nm) operated at 40 mA and 40 kV, with a scan speed of 3°/min having set the 2θ angle 2° to 60°. The crystallinity indices (*C.I*.) of the chitin samples were obtained using following Eq. (1).

1 $${C}.{I}.=({{I}}_{(110)}-{{I}}_{({am})})/{{I}}_{({am})}\times 100$$

Where, I_(110)_ is the maximum intensity at a 2θ ≈ 19° of crystalline material and I_(am)_ is the intensity of amorphous diffraction at a 2θ angle ≈16 °^29^.

FTIR spectra of chitin and chitosan samples were obtained using a Fourier transform infrared (FTIR) spectrometer, VERTEX 70 FTIR (Bruker optik GmbH), with a resolution of 4 cm^−1^ and accumulation of 32 scans between 4000 to 400 cm^−1^ on ATR mode. The degree of deacetylation (DD) of chitosan samples were calculated by comparing the absorbance of the measured peak at 1650 cm^−1^(proportional to the DA) to that of the reference peak at 3450 cm^−1^ (independent of the DA) using following Eq. (2)^19^

2 $${DD}( \% )=100-[({{A}}_{(1650)}-{{A}}_{(3450)})\times 100]$$

### Intrinsic viscosity and molecular weight

Intrinsic viscosity and viscosity-average molecular weight was determined using Ubbelohde capillary viscometer (0.8 mm capillary diameter) on four concentration of chitosan solution in 0.3 M acetic acid+0.2 M Sodium acetate solvent system at 25 °C^30^. The values obtained after experiments were used in the equation of Mark–Houwink and molecular weights were determined using the following Eq. (3)

3 $$[{\eta }]={K}{{M}}^{{\alpha }}$$

Where, [η] is intrinsic viscosity, K and α are Mark-Houwink Constants. The values of K and α are taken from what was previously determined with identical setting as 0.74 ×10^−3^ and 0.76, respectively.

### Mechanical testing

The mechanical properties of chitosan film and FLAM of insect chitin were evaluated by the tensile test performed using UTM (Universal Testing Machine- Instron 5943) equipped with 1 kN load cell with a cross head speed of 4 mm min^−1^ at ambient conditions according to the standard method (ASTM D1037-12)^15^. Chitosan films specimens were cut into strips with 70 mm length and 20 mm width. Thickness of the chitosan film were measured by SEM. The specimens for FLAMs were cast in a dog-bone shape mold with 100 × 16 × 6 mm size in the reduced section and allowed to dry in an oven at 50 °C for 24 h before test. 3-point bending test: FLAM (CC 1:8) were tested for the flexural test following ASTM standards (D1037–12) on UTM equipped with 3-point flexure test fixtures. A support span of 60 mm and head speed of 4 mm min^−1^ was used.

### Figures

Figures and clip art compositions were prepared using Adobe Illustrator (Adobe Inc, San Jose, California, U.S.). Photographs were taken using a Canon EOS 50D camera (Canon Inc. Ota City, Tokyo, Japan) with 18–55 mm lens.

## Supplementary information

Supplementary information.

Supplementary information2.

## Data Availability

All relevant data supporting the key findings of this study are available within the article and its Supplementary Information files or from the corresponding authors upon reasonable request.
