# Supplementary material for: Circular manufacturing of chitinous bio-composites via bioconversion of urban refuse
Source: Sci Rep. 2020 Mar 13;10:4632. doi: 10.1038/s41598-020-61664-1 (PMC7070007; doi:10.1038/s41598-020-61664-1)
Supplement: Supplementary file 1 — Supplementary information. [file 41598_2020_61664_MOESM1_ESM.pdf]

Supporting Online Material

Circular manufacturing of chitinous bio-composites via bioconversion of urban refuse

Naresh D. Sanandiya, Christoph Ottenheim, Phua Jun Wei, Augusta Caligiani, Stylianos Dritsas, Javier G. Fernandez*

*To whom correspondence should be addressed.
E-mail: Javier.fernandez@sutd.edu.sg

This material includes:

Additional Discussion 3D printing setup and technology characteristics

Table 1 Intrinsic viscosity and molecular weights of chitosan
Table 2 Assignment of the FTIR bands to the respective vibrations of chitin from the *H. illucens of two different geological locations*
Table 3 Assignment of the FTIR bands of chitosan from the *H. illucens*

Figure 1 Flow chart of the chitin and chitosan extraction from *H. illucens*
Figure 2 FTIR spectra of (a)chitin and (b)chitosan from *H. illucens of two different geographical locations*
Figure 3 SEM images of cross-section of CS films and thickness measurement
Figure 4 Additional examples of geometries 3D printed
Figure 5 High Magnification images of 3D printed layers

Movie 1 3D printing of scaled Hydra with a 0.5mm filament

3D Printing Setup and Technology Characteristics

Description: The additive manufacturing setup includes a six-axis industrial articulate robot (fine-positioner), mounted on top of the scissor lift mobile platform (coarse-positioner), a bulk unloading pump for adhesives and sealants (material supply), transported mechanically via a hose to an auger extruder (volumetric dispenser) mounted on the robot's flange.

Positioning: The coarse-fine positioning strategy is used for expanding the build-volume without the requirement for a proportionally large cartesian gantry system, such as those often used in small-scale rapid prototyping systems and large-scale concrete printers. For small-scale applications, a cartesian system can be used with insignificant modifications, i.e., simplification of the motion control system. The theoretical static reach of the robot, at its flange, is 1.65m horizontally and 3.7m vertically. With the reposition of the mobile robot platform and calibration update, the work envelope can be extended as required.

Dispensing: The additive manufacturing method is formally Material Extrusion as per ISO/ASTM 52900, combining principles from Fused Deposition Modeling and Direct Ink Writing. FLAM is an adhesive deposited in wet-state at room temperature and cures by drying over time, either at ambient conditions or by forced convection. The maximum rated flow rate of the system is 3.5ml/sec. The range of nozzle diameters used, within the same system, ranging from 1.0mm to 7.0mm, with proper material flow and linear motion coordination.

Printing: In large-scale mode, the maximum 3D printing velocity achieved is approximately 50mm/sec with 3.5ml/sec, 6.5mm nozzle, with limiting factor being the upper bound of the pump's pressure rating. In small-scale mode, the maximum velocity reached is circa 150mm/sec, 0.05ml/sec, 1.5mm nozzle size, with limiting factor being motion-induced vibration. Indicatively, a typical PLA (1.12g/cm^3) FDM 3D printer dispenses circa 0.005ml/sec at 50mm/sec with a 0.2mm nozzle at 200°C. Here, because energy input and temperature control are limiting neither material flow rate nor linear velocity, we are able to extrude at orders of magnitude higher compared to thermoplastics.

References: Additional literature regarding additive manufacturing of FLAM.

1 Dritsas, S. *et al.* Additive Manufacturing with Natural Composites-From material intelligence to informed digital fabrication. (2019).

2 Vijay, Y., Sanandiya, N. D., Dritsas, S. & Fernandez, J. G. Control of Process Settings for Large-Scale Additive Manufacturing With Sustainable Natural Composites. *Journal of Mechanical Design* **141**, doi:10.1115/1.4042624 (2019).

3 Dritsas, S. & Soh, G. S. Building robotics design for construction. *Construction Robotics* **3**, 1-10 (2019).

4 Dritsas, S., Vijay, Y., Dimopoulou, M., Sanadiya, N. & Fernandez, J. G. in *Robotic Fabrication in Architecture, Art and Design.* 181-191 (Springer).

Table 1. Intrinsic viscosity and viscosity-average molecular weight of chitosan

Sample	*Intrinsic viscosity (dL/g)*	*Molecular weight*
H. illucens (SG)	6.43±0.032	152450.7±994.90
H. illucens (IT)	5.97±0.019	138198.2±605.44
P. monodon	7.59±0.036	189520.5±1179.19

Data presented are mean of triplicate measurements (±SD)

Table 2. Assignment of the FTIR bands to the respective vibrations of chitin from the *H. illucens of two different geological locations*

Assignments	Wavenumber (cm^{-1})	
	H. illucens (SG)	*H. illucens* (IT)
ν(OH)HB	3432	3436
ν(NH_2)	3257	3256
ν(CH_3)	2927	2924
ν(CO) Am I	1652	1653
δ(NH2)	1619	1618
Am II	1550	1550
δ(CH_3) + δ(CH)	1415	1470
δ(CH_2) + δ(CH) + δ(OH) +ν(ϕ)	1373	1375
δ (CH) +ν (CN)	1305	1307
ν (COC) + ν(ϕ)	1153	1153
ν (COC) + ν(ϕ)	1067	1069
ν(ϕ) + δ (CH)	1007	1009
ν (CN)	949	951
ν(ϕ)	892	894

ν, stretching; δ, in-plane bending vibration; ϕ, pyranoid ring; HB, hydrogen bond

Table 3. Assignment of the FTIR bands to the respective vibrations of chitosan from the *H. illucens*

Assignments	Wavenumber (cm^{-1})	
	H. illucens (SG)	*H. illucens* (IT)
ν(OH)HB + ν(NH_2)	3361	3362
ν(CH_3)	2921	2920
ν(CH_2)	2853	2851
ν(CO)	1650	1650
δ(NH_2)	1582	1586
δ(CH) + δ(OH)	1420	1420
δ(CH_2) + δ(CH) + δ(OH) +ν(ϕ)	1377	1378
δ (CH) +ν (CN)	1323	1322
δ(OH…O) + ν(C-C) + ν(C-O)	1248	1250
ν (COC) + ν(ϕ) + ν(C-OH) + ν(C-CH_2)	1148	1148
ν (COC) + ν(ϕ) + ν(C-OH) + ν(C-CH_2)	1056	1056
ν(ϕ) + δ (CH)	1028	1029
ν (CN)	987	988
ν(ϕ)	889	890

ν, stretching; δ, in-plane bending vibration; ϕ, pyranoid ring; HB, hydrogen bond

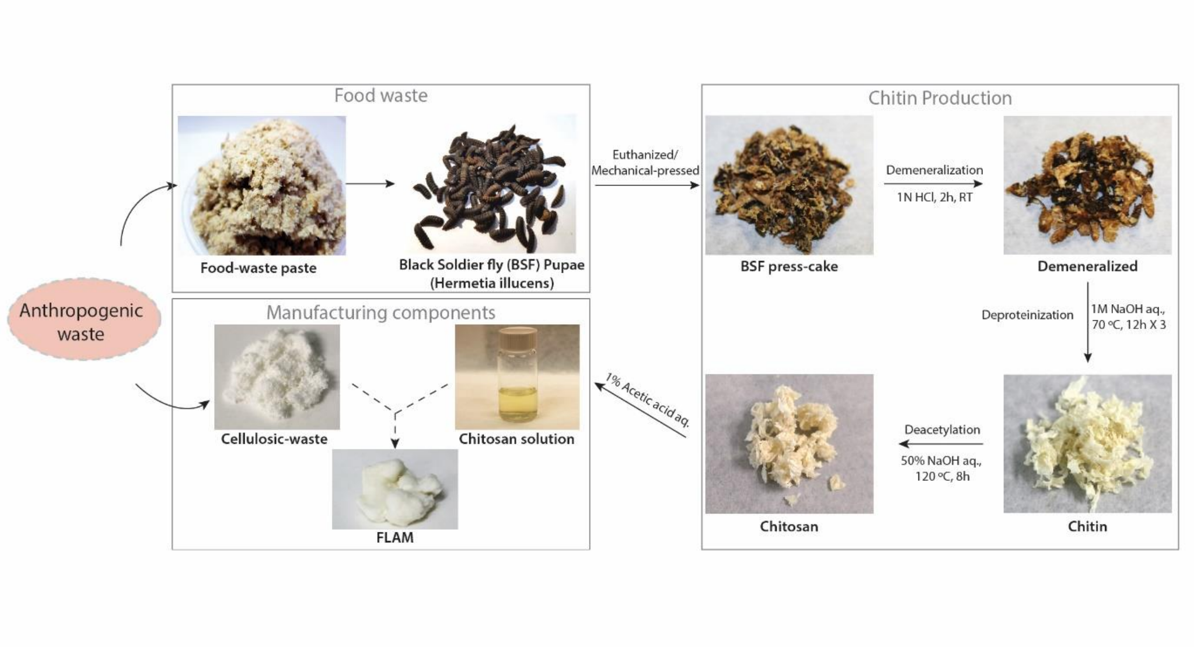

Supplementary Figure 1 | Flow chart of the chitin and chitosan extraction from *Hermetia illucens*

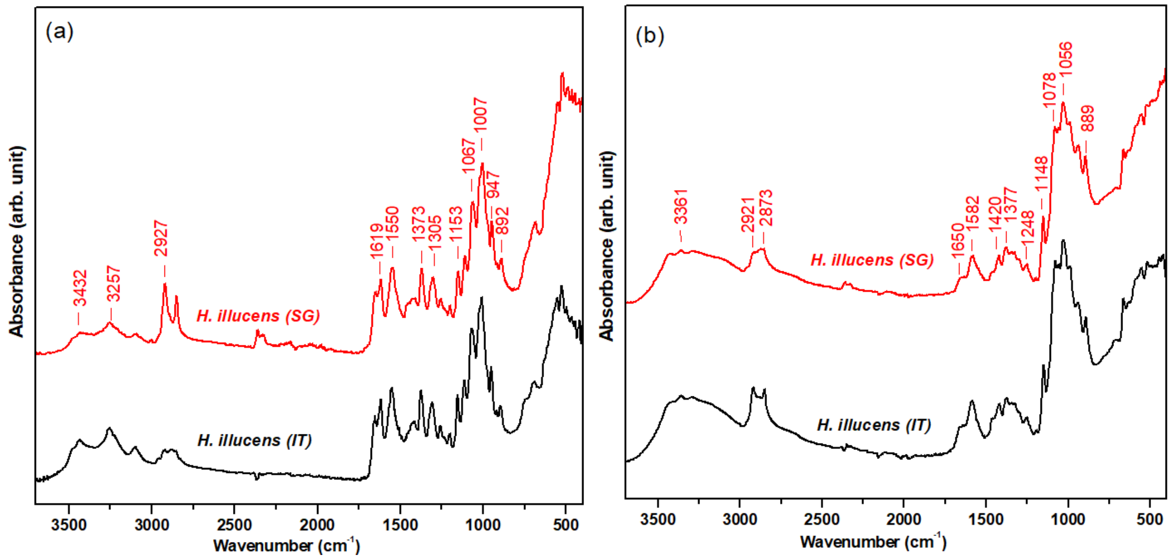

Supplementary Figure 2 | FTIR spectra of (a)chitin and (b)chitosan from *H. illucens of two different geographical locations*

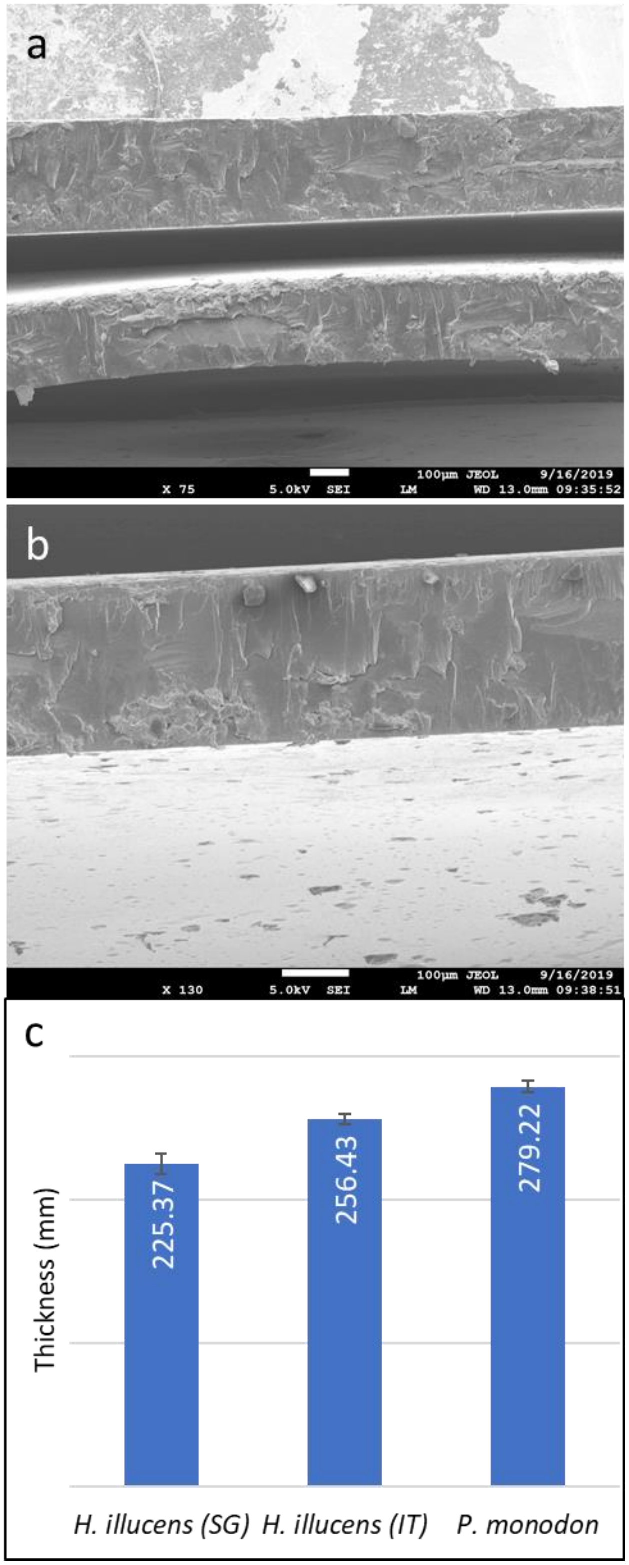

Supplementary Figure 3 | SEM images of cross-section of CS films from (a) P. monodon, top; *H illuccens*(SG), bottom, (b) *H. illucens*(IT) and (c) thickness measurement

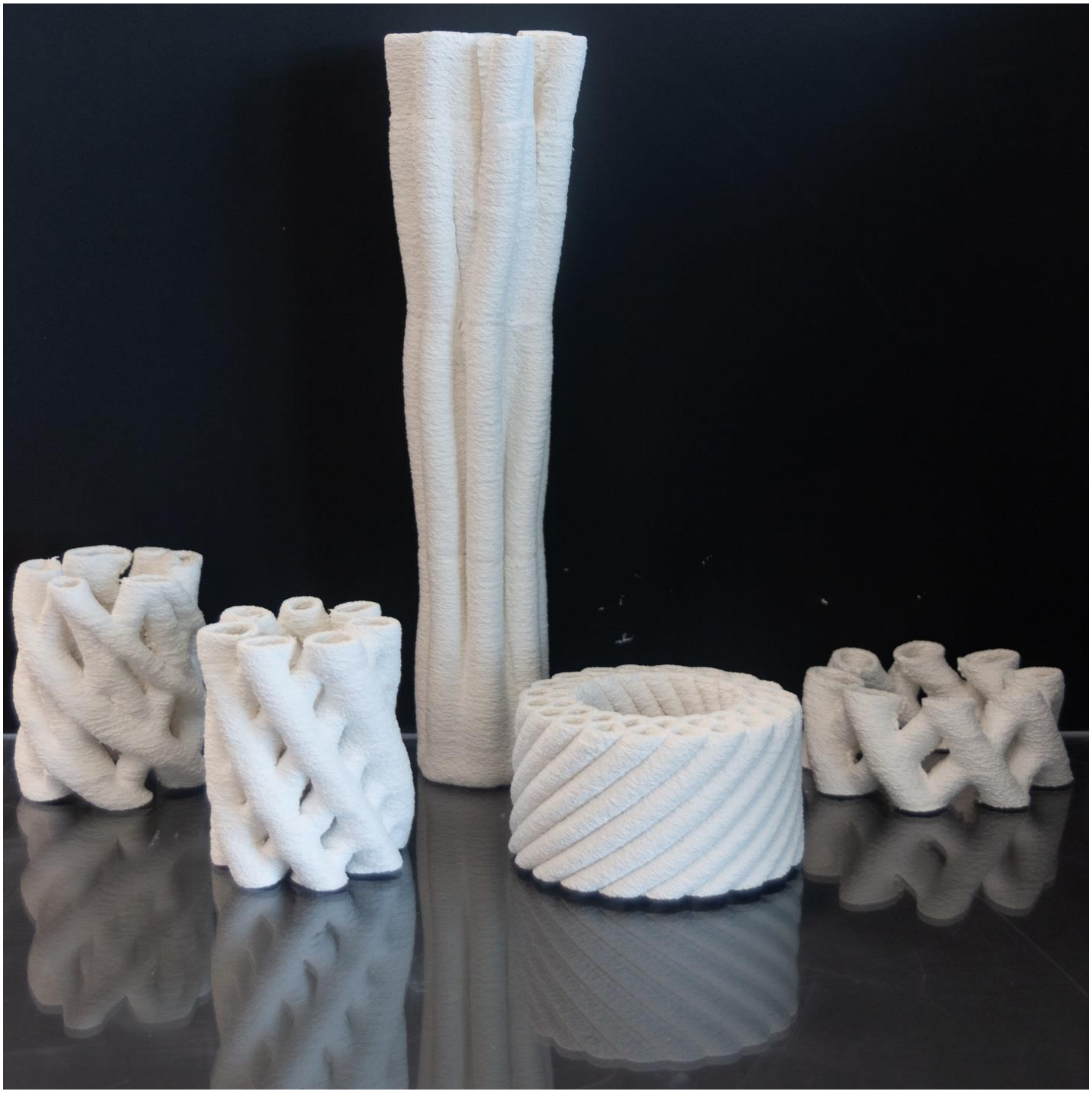

Supplementary Figure 4 | Additional examples of geometries 3D printed with FLAM and unable to be produced by traditional casting or molding techniques.

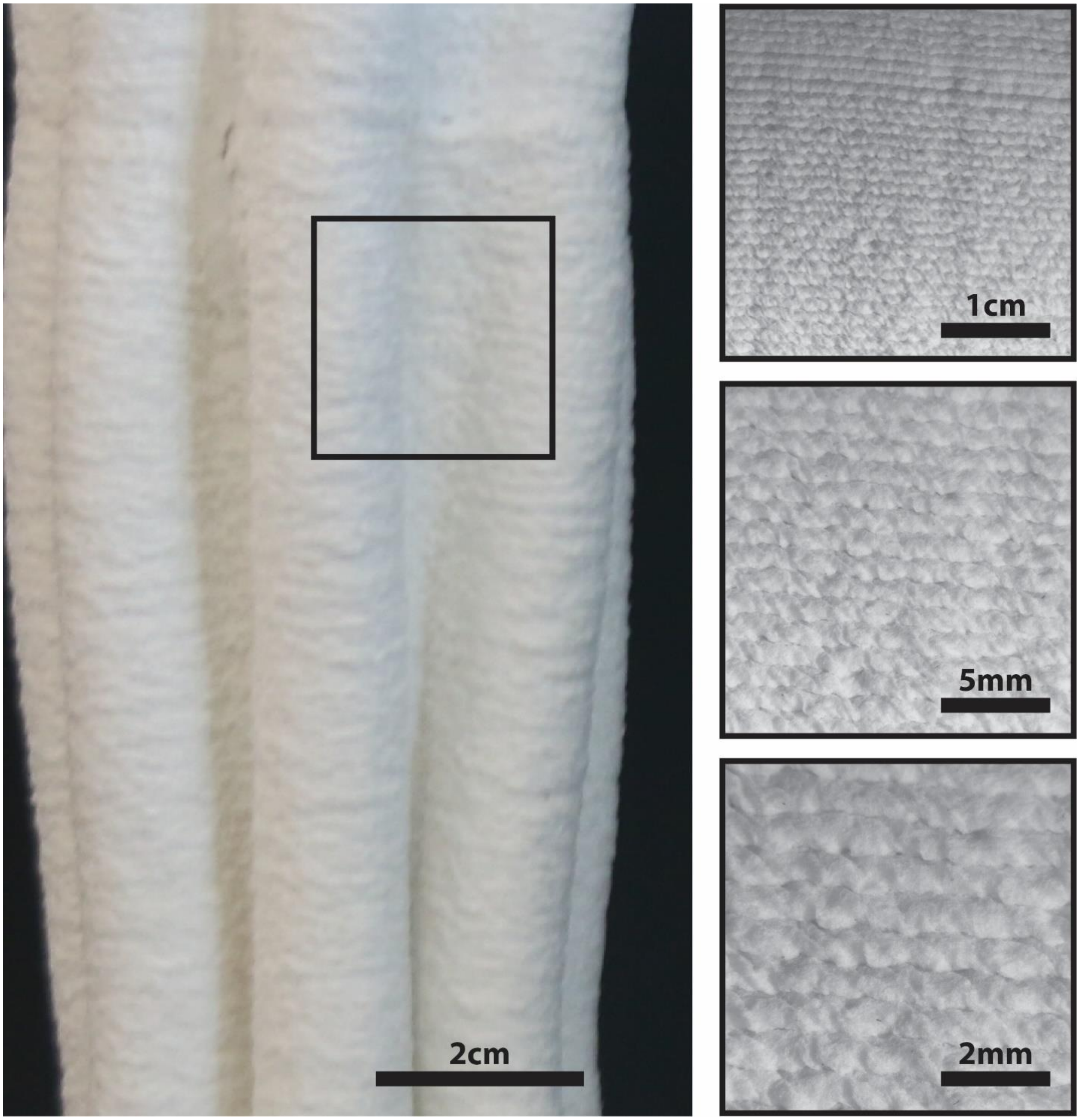

Supplementary Figure 5 | High magnification images of the urban Hydra in Figure 4 on the main text, and Supplementary Figure 4. High magnification images show the microstructured layered surface resulting from the 3D printing process.
